# White Blood Cell and Granulocyte Counts Are Independent Predictive Factors for Prognosis of Advanced Pancreatic Caner

**DOI:** 10.1155/2018/8096234

**Published:** 2018-05-08

**Authors:** Lanyun Feng, Shihui Gu, Peng Wang, Hao Chen, Zhen Chen, Zhiqiang Meng, Luming Liu

**Affiliations:** ^1^Department of Integrative Oncology, Fudan University Shanghai Cancer Center, Shanghai, China; ^2^Department of Oncology, Shanghai Medical College, Fudan University, Shanghai, China; ^3^Department of Hand Surgery, Huashan Hospital, Fudan University, Shanghai, China; ^4^Key Laboratory of Hand Reconstruction, Ministry of Health, Shanghai, China; ^5^Shanghai Key Laboratory of Peripheral Nerve and Microsurgery, Shanghai, China

## Abstract

**Background:**

Pancreatic cancer is associated with high death rates and limited therapeutic options, with no effective predictive factors being available for prognosis at present. In this study, we evaluate the value of using blood test results for pancreatic cancer prognosis.

**Method:**

The records of 214 pancreatic cancer patients were reviewed. Blood test results for white blood cell (WBC), granulocyte, neutrophil, lymphocyte, platelet count, neutrophil-to-lymphocyte ratio (NLR), and platelet-to-lymphocyte ratio (PLR) were dichotomized on the basis of median values. This was followed by univariate and multivariate analyses between groups.

**Results:**

Patients with pretreatment values in the range WBC ≥ 5.8 × 10^9^/L, granulocyte ≥ 3.7 × 10^9^/L, neutrophil ≥ 3.9 × 10^9^/L, lymphocyte < 1.4 × 10^9^/L, and NLR ≥ 2.8 showed significant correlations pointing to poorer overall survival. Multivariate analysis indicated that WBC ≥ 5.8 × 10^9^/L (HR = 1.808; 95% CI = 1.055–3.096; *p* = 0.031) and granulocyte ≥ 3.7 × 10^9^/L (HR = 7.346; 95% CI = 1.275–42.321; *p* = 0.026) can be taken to be independent prognostic factors for overall survival in pancreatic patients.

**Conclusion:**

Pretreatment values of WBC and granulocyte count were independent factors with poor prognosis ability with respect to pancreatic cancer.

## 1. Introduction

Pancreatic cancer is one of the most fatal cancers. It is ranked the fourth leading cause of cancer deaths in the United States for both sexes [1]. In China, the incidence of pancreatic cancer was noted to be 90,100 with a mortality rate of about 79,400 per year in 2015 [2]. Pancreatic cancer cases are usually diagnosed only in an advanced stage. Also, it is associated with limited available effective treatment options. For these reasons, the 5-year survival rate of pancreatic cancer is below 5%. For patients suffering from advanced pancreatic cancer, the Karnofsky performance status (KPS) usually gets worsened, so effective treatment and precise selection of patients are of great importance. Currently, feasible prognosis prediction methods are still lacking for this ailment. Although pathological classification could be used as a good predictor, it is not easy to harvest the tumor tissue for biopsy in most of the patients.

However, certain recent blood test results provide us with clues for cancer prognosis prediction. These results include white blood cell counts, neutrophil counts, lymphocyte counts, granulocyte counts, neutrophil-to-lymphocyte ratios (NLRs), and platelet to lymphocyte ratios (PLRs). These parameters reflect the inflammatory status of the patient, which may play decisive roles at different stages as the tumor progresses. The stages include initiation, promotion, invasion, and metastasis [3, 4]. Both innate (including macrophages, neutrophils, mast cells, myeloid-derived suppressor cells, dendritic cells, and natural killer cells) and adaptive (T and B lymphocytes) immune cells exist in the tumor's microenvironment. These immune cells participate in inflammatory responses within the microenvironment of the tumor. The consequences of inflammation in cancer are still under debate. On the one hand, inflammation may promote tumor progression [5]. On the other hand, it enhances tumor antigen cross-presentation and subsequently induces antitumor immune responses [6].

Neutrophil is the most common and vital element of inflammation that plays a significant role influencing the microenvironment of the tumor [7]. NLR is a general inflammatory indicator that can be estimated by dividing the neutrophil count by the lymphocyte count. NLR has been proposed as a predictive factor for different types of carcinomas, such as metastatic melanoma [8], esophageal cancer [9], colorectal cancer [10], non-small-cell lung cancer (NSCLC) [11], metastatic castration-resistant prostate cancer (mCRPC) [12], diffuse large B-cell lymphoma (DLBCL) [13], and pancreatic cancer [14]. PLR is another systemic inflammation index that can be calculated by dividing the circulating platelet count by the lymphocyte count. PLR has been shown to take on prognostic roles in several cancers, for example, breast cancer [15], NSCLC [16], and nasopharyngeal cancer [17]. However, the predictive roles of these factors in pancreatic cancer patients are still controversial.

The prognostic role of the inflammatory factors in patients with pancreatic cancer are evaluated in the present study, which aims at coming up with clues facilitating the prognosis of pancreatic cancer by using results from routine laboratory tests.

## 2. Materials and Methods

### 2.1. Patients

Patients with pancreatic cancer who had visited our department at Fudan University Shanghai Cancer Center (FUSCC) between January 2012 and January 2014 were included in this retrospective study. Diagnoses of pancreatic cancer for patients who had received surgery before but had experienced a recurrence or metastasis were performed through pathological examinations of the respective surgical samples. For patients who could not tolerate surgical treatment in their first visit to doctors, the diagnosis was confirmed by pathological biopsy. The study was approved by the ethics committee of FUSCC. Informed consent was acquired from each patient. All relevant blood tests were performed in accordance with institutional guidelines and regulations. Patient characteristics were extracted from their medical records. The characteristics included sex, age, location of the pancreas cancer, the TNM stage, outcomes, and the blood test—white blood cell (WBC), granulocyte, neutrophil, lymphocyte, and platelet—results at the time of diagnosis. The neutrophil-to-lymphocyte ratio (NLR) and platelet-to-lymphocyte ratio (PLR) values were calculated. The primary endpoint was overall survival (OS) as calculated from the date of first diagnosis to death or the last follow-up. Patients with no data, no pathological diagnoses, no informed consent, or poor treatment compliance were excluded. Patients were followed up every six months until death or loss of follow-up.

### 2.2. Statistical Analysis

All quantitative data were presented as SEM or median (range) as specified. The quantitative data were separated using median values and analyzed using Prism GraphPad (version 5, La Jolla, CA) and SPSS software (version 19.0, Armonk, NY). The Kaplan–Meier method was applied while conducting univariate analyses. Obvious differences between groups were determined using log-rank tests. The Cox proportional hazard model was used for multivariate analysis. Hazard ratios (HRs) were reported as relative risks along with the 95% confidence intervals (CIs). All tests were 2-sided, and *p* < 0.05 was taken as the criterion indicating statistical significance.

## 3. Results

214 patients who were pathologically diagnosed with pancreatic cancer were included. The records of these patients were retrieved and analyzed. The median age of all patients was 61 (age range: 27–85). 36.0% of them were female (77/214). Almost 38.8% (83/214) of patients were categorized as TNM stage 3, and 61.2% (131/214) of patients were at TNM stage 4 (Table 1). The WBC count was (6.4 ± 0.2) × 10^9^/L, median value 5.8 (range: 1.7–20.3) × 10^9^/L; granulocyte count (4.3 ± 0.1) × 10^9^/L, median value 3.7 (range: 0.7–18.3) × 10^9^/L; neutrophil count (4.2 ± 0.2) × 10^9^/L, median value 3.9 (range: 0.9–17.9) × 10^9^/L; lymphocyte count (1.5 ± 0.0) × 10^9^/L, median value 1.4 (range: 0.3-4.2 × 10^9^/L); and platelet count (180.1 ± 5.1) × 10^9^/L, median value 170 (range: 27–543) × 10^9^/L. The NLR was 3.5 ± 0.2, with a median value of 2.8 (range: 0.6–22.8). The PLR value was 135.9 ± 4.9, with a median value of 124.6 (range: 30–604). Please see Table 2 for full details.

**Figure 1:**
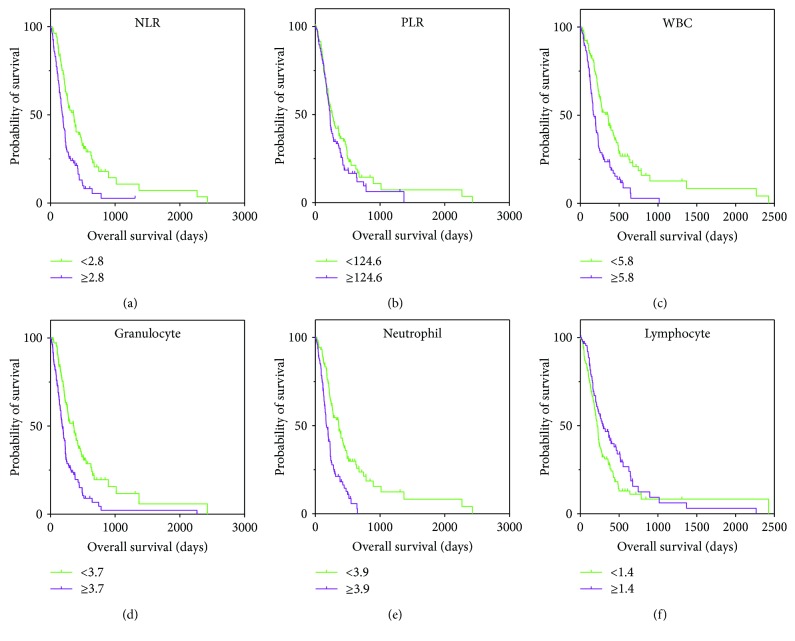
Kaplan–Meier survival curves for overall survival (OS) in patients with pancreatic cancer after diagnoses. (a) The OS of NLR < 2.8 was longer than that of NLR ≥ 2.8 (*p* < 0.0001, log-rank). (b) The OS of patients with PLR < 124.6 was not significantly different from those with PLR ≥ 124.6 (*p* = 0.182, log-rank). (c) The OS of patients with baseline WBC < 5.8 × 10^9^/L was longer than in those with WBC ≥ 5.8 × 10^9^/L (*p* < 0.0001, log-rank). (d) The OS of patients with baseline granulocyte < 3.7 × 10^9^/L was longer than in those with granulocyte ≥ 3.7 × 10^9^/L (*p* < 0.0001, log-rank). (e) The OS of patients with baseline neutrophil < 3.9 × 10^9^/L was longer than in those with neutrophil ≥ 3.9 × 10^9^/L (*p* < 0.0001, log-rank). (f) The OS of patients with baseline lymphocyte < 1.4 × 10^9^/L was longer than in those with WBC ≥ 1.4 × 10^9^/L (*p* = 0.034, log-rank).

The prognostic roles of blood test parameters were investigated using univariable and multivariable analyses. The univariable analyses indicated that patients with baseline NLR ≥ 2.8 (*P* < 0.0001), WBC ≥ 5.8 × 10^9^/L (*P* < 0.0001), g*r*anulocyte ≥ 3.7 × 10^9^/L (*P* < 0.0001), neutrophil ≥ 3.9 × 10^9^/L (*P* < 0.0001), lymphocyte < 1.4 × 10^9^/L (*p* = 0.034) had poor OS figures for this study cohort. However, age, gender, location of carcinoma, TNM classification, and PLR were not significantly associated with the prognosis (Table 3 and Figure 1).

In multivariate analysis, the Cox proportional hazard model was performed. The analysis revealed that WBC ≥ 5.8 × 10^9^/L (HR = 1.808; 95% CI 1.055–3.096; *p* = 0.031) and granulocyte ≥ 3.7 × 10^9^/L (HR = 7.346; 95% CI 1.275–42.321; *P* = 0.026) were independent prognostic factors for OS in pancreatic cancer patients. However, age, gender, location of carcinoma, TNM classification, neutrophil, lymphocyte, NLR, and PLR were not independent factors for the prognosis of pancreatic cancer (Table 4).

## 4. Discussion

In view of low survival rates and disappointing therapeutic options for pancreatic cancer, many researchers have searched for applicable prognostic factors and clinical indices that are significantly related to pancreatic cancer survival. It has been reported that inflammation and nutritious status are closely related to certain malignant tumors. Monitoring of inflammation status can be easily achieved at a clinic using laboratory testing for WBCs, neutrophils, granulocytes, and so on Therefore, in the present study, we tried to get some clues from routine blood test results for the prognosis of pancreatic cancer.

In our study cohort, the baseline values of WBC, granulocyte, neutrophil, lymphocyte, and NRL were associated with the overall survival of pancreatic cancer patients. However, our multivariate analyses indicated that only the baseline values of WBC and granulocyte count could be used as independent predictive factors for pancreatic cancer. High values of WBC and granulocyte test may indicate poor survival. Likewise, it has been reported that the leukocyte, NLR, and other indicators may reflect systemic inflammation [18–20]. Neutrophils can promote angiogenesis and repress the immune system from supporting tumor growth [3, 21]. The vascular endothelial growth factor (VEGF) and matrix metalloproteinase (MMP), important cytokines and chemokines for tumor microenvironment angiogenesis and metastasis, are believed to be secreted by neutrophils [22, 23]. It has been suggested that increased neutrophil counts are associated with greater tumor sizes and poorer survival rates in patients with confined nasopharyngeal cancer [24] and renal cell carcinoma [25]. Since neutrophils constitute the majority population of granulocytes, which is a main component of WBC, it is reasonable to expect that the increase of WBC in this study was mainly due to the increase of granulocytes, which in turn was a consequence of an increase in neutrophil count.

In the present study, lymphocyte count was negatively associated with survival rate. Reduced lymphocyte counts predict poor survival rates. These results are consistent with other observations [26]. It is widely accepted that lymphocytes play an important role in determining immune responses against tumor. High lymphocyte values result in cytotoxic cell death and inhibit tumor cell proliferation and migration [21, 27]. In patients with carcinoma, the programmed death 1 or programmed death 1 ligand (PD-1/PD-L1) signaling the microenvironment of tumor may compromise the cytotoxic capability of T lymphocytes. In pancreatic cancer patients, high PD-1 expression level on CD8^+^ T lymphocytes is associated with the poor overall survival rate as well as the disease-free survival rate [28]. In patients with pancreatic ductal adenocarcinoma (PDA), PD-L1 negative and HLA class 1 high expression may predict a better prognosis [29]. The activation of PD-1/PD-L1 pathways inhibits the infiltration of cytotoxic T lymphocytes into tumor tissue to kill cancerous cells. This inability of lymphocytes contributes to the survival and progression of tumor cells [30]. Meanwhile, the neutrophil may also suppress the cytotoxic activity of lymphocytes [31]. Our study has shown that decreased lymphocytes are correlated with poor prognosis, a finding that is a consistent observation in previous studies.

In our study, the TNM classification was not associated with the prognosis of the disease. This is different from others' observations [32, 33]. This is probably due to the extremely long survival times of three stage 4 patients, which were 1369, 2226, and 2427 days, respectively.

This study has some limitations. First of all, it was a single-center study with a relatively small sample size. Further, the subjects were all Chinese. This may have limited the generalizability of its findings. Second, due to concealed manifestations, the vast majority of patients are diagnosed at an advanced stage. Therefore, we did not enroll the early-stage patients in view of the very small sample size they would constitute. In addition, our study was retrospective and the exposure data were collected prior to the initiation of the study. Therefore, C-reactive protein (CRP) was not evaluated by us although it is an objective inflammation marker and has been found to be a prognostic indicator in diffuse large B-cell lymphoma [34, 35]. A multicenter prospective study with a larger sample size is advisable in the future for improving the generalizability of our findings.

## 5. Conclusion

Pretreatment WBC and granulocyte alone could be used as an independent factor for pancreatic cancer prognosis. The existence of inflammation in pancreatic cancer may indicate poor survival. Our study has pointed to a feasible way for the prognosis of pancreatic cancer from routine and simple laboratory test results.

## Figures and Tables

**Table 1:** Basic characteristics of 214 pancreatic cancer patients.

Characteristics	*N* (%) (*n* = 214)
Gender	
Female	77 (36.0%)
Male	137 (64.0%)
Age	
≤60	101 (47.2%)
>60	113 (52.8%)
Location	
Head	81 (37.9%)
Body/tail	133 (62.1%)
TNM classification	
Stage 3	83 (38.8%)
Stage 4	131 (61.2%)

**Table 2:** Baseline blood test results of the 214 pancreatic patients included.

Category	Mean (SD)	Median (range)
WBC (× 10^9^/L)	6.4 (0.2)	5.8 (1.7–20.3)
Granulocyte (× 10^9^/L)	4.3 (0.1)	3.7 (0.7–18.3)
Neutrophil (× 10^9^/L)	4.2 (0.2)	3.9 (0.9–17.9)
Lymphocyte (× 10^9^/L)	1.5 (0.0)	1.4 (0.3-4.2)
Platelet (× 10^9^/L)	180.1 (5.1)	170 (27–543)
NLR	3.5 (0.2)	2.8 (0.6–22.8)
PLR	135.9 (4.9)	124.6 (30–604)

**Table 3:** Univariate analyses examining the association between clinical characteristics and survival in pancreatic cancer patients.

Characteristics	*N* (%)	HR	95% CI	*p* value
Gender				
Female	77 (36.0%)	1	Reference	
Male	137 (64.0%)	1.121	0.800–1.570	0.508
Age				
≤60	101 (47.2%)	1	Reference	
>60	113 (52.8%)	0.950	0.691–1.305	0.750
Locations				
Head	81 (37.9%)	1	Reference	
Body/tail	133 (62.1%)	1.352	0.965–1.893	0.080
TNM classification				
Stage 3	83 (38.8%)	1	Reference	
Stage 4	131 (61.2%)	0.892	0.314–1.755	0.494
Baseline WBC				
<5.8 × 10^9^/L	107 (50%)	1	Reference	
≥5.8 × 10^9^/L	107 (50%)	2.132	1.524–2.976	<0.0001
Baseline granulocyte				
<3.7 × 10^9^/L	106 (49.5%)	1	Reference	
≥3.7 × 10^9^/L	108 (50.5%)	2.849	2.016–4.016	<0.0001
Baseline neutrophil				
<3.9 × 10^9^/L	104 (48.6%)	1	Reference	
≥3.9 × 10^9^/L	110 (51.4%)	2.488	1.770–3.497	<0.0001
Baseline lymphocyte				
<1.4 × 10^9^/L	123 (57.5%)	1	Reference	
≥1.4 × 10^9^/L	91 (42.5%)	0.710	0.516–0.974	0.034
Baseline NLR				
<2.8	105 (49.1%)	1	Reference	
≥2.8	109 (50.9%)	2.183	1.567–3.040	<0.0001
Baseline PLR				
<124.6	107 (50%)	1	Reference	
≥124.6	107 (50%)	1.241	0.904–1.704	0.182

HR: hazard ratio; CI: confidence interval; WBC: white blood cell; NLR: neutrophil-to-lymphocyte ratio; PLR: platelet-to-lymphocyte ratio.

**Table 4:** Multivariate analyses examining the association between clinical characteristics and survival in pancreatic cancer patients.

Characteristics	*N* (%)	HR	95% CI	*p* value
Gender				
Female	77 (36.0%)	1	Reference	
Male	137 (64.0%)	1.277	0.896–1.821	0.177
Age				
≤60	101 (47.2%)	1	Reference	
>60	113 (52.8%)	0.763	0.549–1.062	0.109
Locations				
Head	81 (37.9%)	1	Reference	
Body/tail	133 (62.1%)	1.384	0.980–1.954	0.065
TNM classification				
Stage 3	83 (38.8%)	1	Reference	
Stage 4	131 (61.2%)	0.888	0.622–1.267	0.512
Baseline WBC				
<5.8 × 10^9^/L	107 (50%)	1	Reference	
≥5.8 × 10^9^/L	107 (50%)	1.808	1.055–3.096	0.031
Baseline granulocyte				
<3.7 × 10^9^/L	106 (49.5%)	1	Reference	
≥3.7 × 10^9^/L	108 (50.5%)	7.346	1.275–42.321	0.026
Baseline neutrophil				
<3.9 × 10^9^/L	104 (48.6%)	1	Reference	
≥3.9 × 10^9^/L	110 (51.4%)	4.682	0.772–28.394	0.093
Baseline lymphocyte				
<1.4 × 10^9^/L	123 (57.5%)	1	Reference	
≥1.4 × 10^9^/L	91 (42.5%)	0.678	0.444–1.037	0.073
Baseline NLR				
<2.8	105 (49.1%)	1	Reference	
≥2.8	109 (50.9%)	1.435	0.913–2.257	0.117
Baseline PLR				
<124.6	107 (50%)	1	Reference	
≥124.6	107 (50%)	1.078	0.752–1.546	0.683

Note: the multivariate Cox regression model adjusted for gender, age, location, TNM classification, WBC, granulocyte, neutrophil, lymphocyte, NLR, and PLR. HR: hazard ratio; CI: confidence interval; WBC: white blood cell; NLR: neutrophil-to-lymphocyte ratio; PLR: platelet-to-lymphocyte ratio.

## Data Availability

The datasets analyzed during the current study are available from the corresponding author on reasonable request.
